# Association of systemic immunity-inflammation index with metabolic syndrome in U.S. adult: a cross-sectional study

**DOI:** 10.1186/s12877-023-04635-1

**Published:** 2024-01-15

**Authors:** Peng Zeng, Cheng Jiang, Anbang Liu, Xinyuan Yang, Feng Lin, Lingli Cheng

**Affiliations:** 1 Department of Cardiology, Shenzhen People’s Hospital, Shenzhen, 518000 China; 2 Shandong First Medical University, Central Hospital Affiliated to Shandong First Medical University, Jinan, 250000 China; 3 The Sixth Affiliated Hospital of Guangzhou Medical University, Qingyuan City People’s Hospital, Qingyuan, 511518 Guangdong China

**Keywords:** Systemic immunity-inflammation index, Metabolic syndrome, Cross-sectional study

## Abstract

**Background:**

Metabolic syndrome (MetS) is a pathological condition characterized by the abnormal clustering of several metabolic components and has become a major public health concern. We aim to investigate the potential link of Systemic immunity-inflammation index (SII) on MetS and its components.

**Methods and result:**

Weighted multivariable logistic regression was conducted to assess the relationship between SII and MetS and its components. Restricted cubic spline (RCS) model and threshold effect analysis were also performed. A total of 6,999 U.S. adults were enrolled. Multivariate model found that SII were positively associated with MetS (OR = 1.18;95CI%:1.07–1.30) and hypertension (OR = 1.22; 95CI%:1.12–1.34) in a dose-dependent manner. When SII was converted into a categorical variable, the risk of MetS increased by 36% and the risk of hypertension increased by 53% in the highest quantile of SIIs. The RCS model confirmed linear associations between SII and MetS, as well as a non-linear association between SII and certain components of MetS, including hypertension, hyperglycemia, low HDL, and hyperlipidemia. Meanwhile, the relationship between SII and hypertension presents a J-shaped curve with a threshold of 8.27, above which the risk of hypertension increases. Furthermore, in MetS and hypertension, age, sex, body mass index (BMI), and race were not significantly associated with this positive association based on subgroup analyses and interaction tests(*p* for interaction > 0.05).

**Conclusions:**

The present study indicated that there was a higher SII association with an increased risk of MetS and hypertension in adults. However, further prospective cohort studies are required to establish a causal relationship between SII and MetS, as well as its components.

**Supplementary Information:**

The online version contains supplementary material available at 10.1186/s12877-023-04635-1.

## Introduction

Metabolic syndrome (MetS) is a clinical condition distinguished by hyperglycemia, dyslipidemia, hypertension, and central obesity, which has rapidly increased in the United States and has affected over one-third of American adults in recent years [1–4]. MetS can significantly increase the risk of cardiovascular disease [5, 6], diabetes [7], and some cancers [8]. Inflammation is considered to be the pathophysiological basis of the various components of MetS [5, 9]. Research suggests that healthy lifestyles, including appropriate exercise, weight loss, smoking cessation, and the Mediterranean diet, along with the use of various medications like aldosterone antagonists, statins, and metformin, may alleviate the progression of MetS partly by targeting different underlying inflammatory mechanisms [10–12]. Clinically, identifying suitable inflammatory biomarkers related to MetS aids in assessing and predicting MetS risk, guiding treatment, and evaluating drug efficacy. Although traditional inflammatory markers like CRP have some value in assessing inflammation status and its association with MetS, they may not provide comprehensive information, as inflammation is a complex physiological process involving various biomarkers and pathways [13, 14]. Therefore, finding more comprehensive inflammation markers is a critical research direction.

Some composite inflammatory indices based on blood cell counts, including Neutrophil-to-Lymphocyte Ratio (NLR), Platelet-to-Lymphocyte Ratio (PLR), and Systemic immunity-inflammation index (SII), may effectively reflect the intricate inflammatory conditions within the organism [15, 16]. Among these, SII incorporates the levels of three inflammatory cell types, namely Neutrophil Count (NC), Lymphocyte Count (LC), and Platelet Count (PC). Relative to NLR and PLR, which focus on specific ratios of certain cell types, SII is capable of reflecting the interactions of multiple cell types, providing a more comprehensive response to the complex immune-inflammatory status of the organism. Additionally, by considering a broader spectrum of inflammation-related cells, SII has the potential to mitigate the influence of individual variations, dietary factors, and medications, leading to improved predictive stability [17, 18]. Moreover, some studies have reported that SII may offer a more reliable prediction of disease progression and outcomes in certain inflammation-related diseases, such as cardiovascular diseases [19, 20], cancer [21–23], and metabolic disorders [24, 25]. Furthermore, considering the accessibility and affordability of SII in community healthcare settings, it holds promise as an effective tool for predicting the risk of MetS. The association between SII and MetS, as well as its individual components, remains incompletely elucidated due to the scarcity of available research. The main objective of this study was to investigate the relationship between MetS and its individual components, as well as SII, in a sample of adult participants from the National Health and Nutrition Examination Survey (NHANES). Based on previous empirical evidence, we can hypothesize that there is a positive correlation between SII and MetS, as well as its components.

## Methods

### Study design

**Fig. 1:**
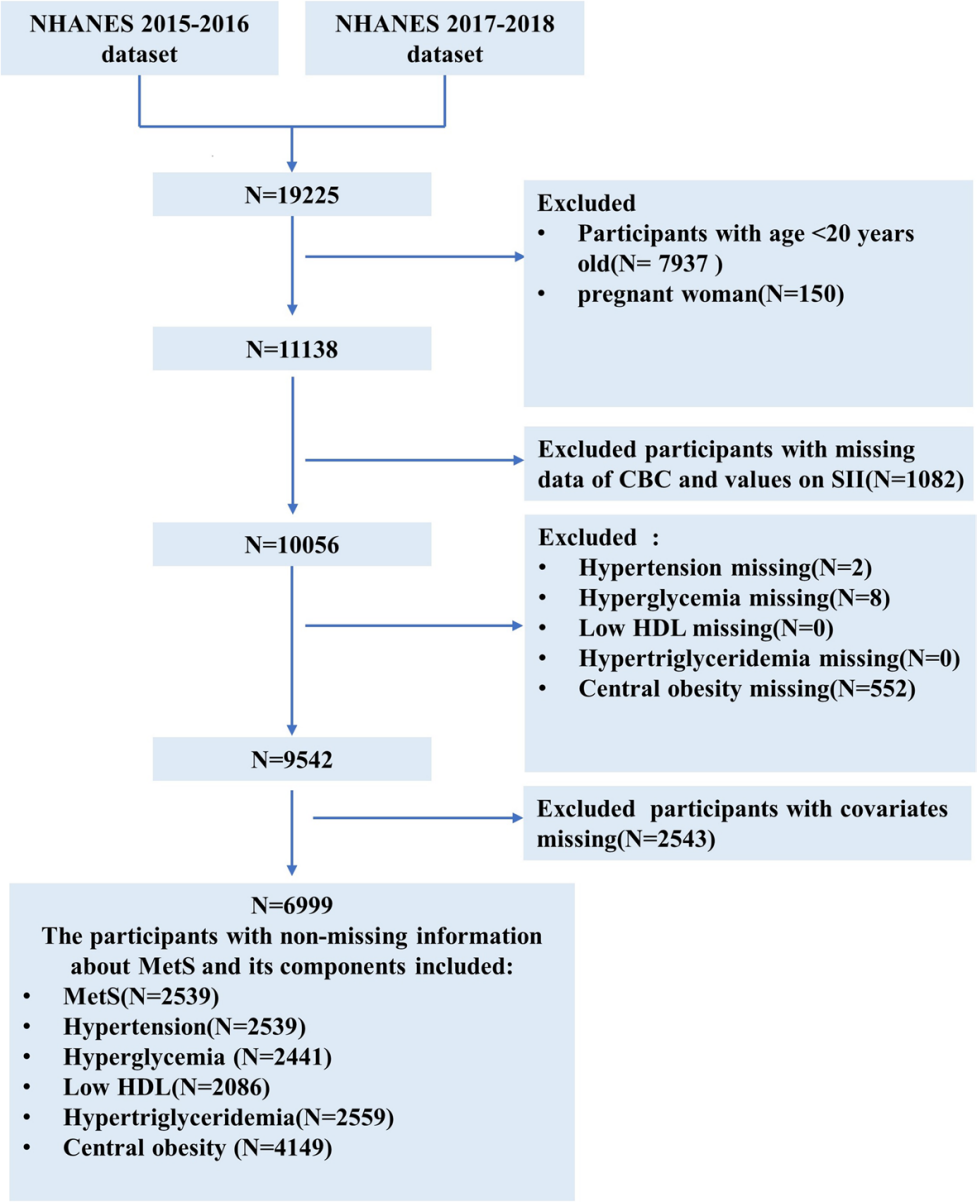
Flowchart of the population included in our final analysis

The NHANES is an ongoing research initiative that aims to evaluate the overall health and nutritional well-being of the American population through a representative cross-sectional sample. Detailed datasets and additional information can be found on the NHANES website [26]. We extracted data from NHANES (2015–2018), with U.S. Adults (age ≥ 20 years) interviewed (Fig. 1). To minimize the introduction of estimation errors as much as possible, we opted to utilize the complete case analysis method [27]. This involved the exclusion of observations that contained any missing information, including incomplete data on complete blood count tests, unavailable data regarding the diagnosis of MetS, and incomplete data for other potential confounding factors.

### Assessment of MetS and its components

According to the NCEP-ATP III criteria [4], MetS is diagnosed if it includes at least the following three components:1. central obesity: Waist circumference (men ≥ 102 cm, women ≥ 88 cm); 2. Hypertriglyceridemia: Serum triglycerides ≥ 150 mg/dL; 3. Low high-density lipoprotein cholesterol (low HDL): Serum high-density lipoprotein cholesterol (HDL-C) levels < 40 mg/dL for men and < 50 mg/dL for women; 4. Hypertension: Systolic blood pressure ≥ 130 mmHg or diastolic blood pressure ≥ 85 mmHg or currently using antihypertensive medication or diagnosed with hypertension by a physician; 5 Hyperglycemia Fasting blood glucose ≥ 100 mg/dL or currently receiving glucose-lowering therapy or diagnosed with diabetes. Information on medication use and disease diagnosis was collected from participants through self-reported questionnaires and interviews. The systolic and diastolic blood pressure values for all participants were calculated as the arithmetic mean of repeated measurements (up to 4 times).

### SII and covariate

**Fig. 2:**
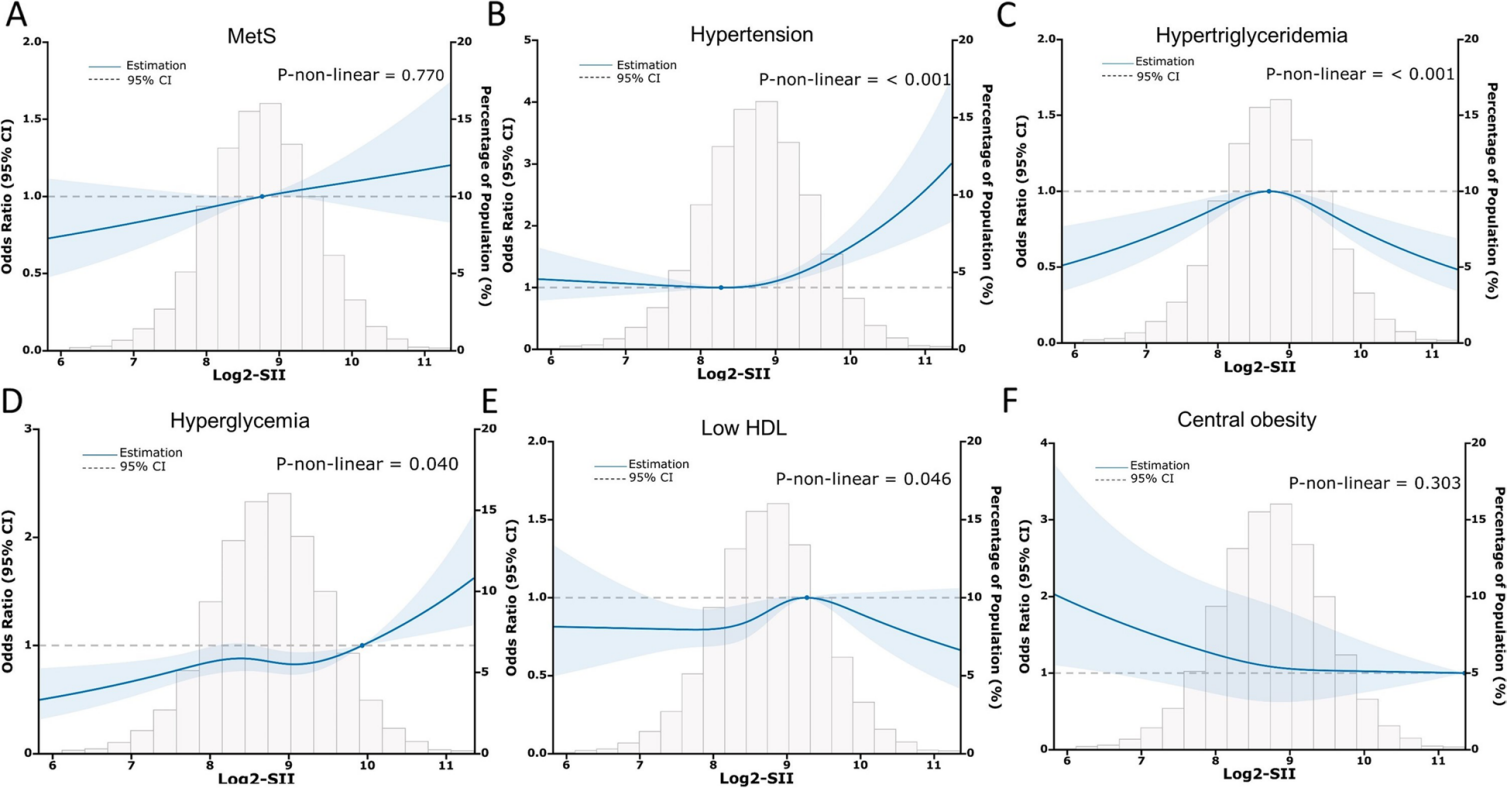
Association between SII and MetS and its components risk

SII was calculated as PC * (NC/ LC), utilizing the data obtained from the complete blood count analysis [15, 23].To account for the right-skewed distribution of SII, a log2 transformation (log2-SII) was applied to approximate a normal distribution (Fig. 2). The analysis included potential confounding factors related to SII and MetS based on previous studies [28].The study incorporated various continuous variables such as age, minutes sedentary activity, serum uric acid (SUA) levels, serum creatinine [29] levels, and blood urea nitrogen (BUN) levels. Additionally, categorical variables such as sex, race, education level, body mass index (BMI), physical activity level, drinking status, and smoking behavior poverty-to-income ratio [30], marital status, were also considered.

### Statistical analysis

The statistical analysis in this study utilized the mobile examination center exam weight by NHANES protocol [31]. Descriptive analysis was conducted by calculating the mean (standard deviation, SD) or median (interquartile range, IQR) for continuous variables and the frequency for categorical variables. A weighted multivariable logistic regression analysis was then performed to examine the relationship between SII and MetS, as well as its individual components. Using the change in estimations principle for variable selection, we eliminated variables that had an effect on the model of less than 10%. The analysis was adjusted for various factors including age, sex, race, PIR, education, drinking status, smoking status, BMI, physical activity, sedentary activity, CR, BUN, and SUA. Odds ratios (OR) and 95% confidence intervals (CIs) were used to assess the risk of MetS or its components [32]. SII was converted into a categorical variable, and P for trend was calculated. Non-linear associations between SII levels and MetS and its components were examined using restricted cubic splines (RCS) and likelihood ratio tests [33]. The reference value for RCS was determined based on the shape of the curve. Subgroup analysis was performed by age, sex, race, and BMI. A significance level of *P* < 0.05 was considered statistically significant. All statistical analyses were performed using R version 4.2.2.

## Result

Our analysis consisted of a cohort of 6999 adult participants, which was a representative sample of 167,186,185 individuals when weighted. Among the sample, 3425(48.93%) were male and the average age was 47.32 ± 16.68 years old. Table 1 provides an overview characteristics of participants by MetS. Approximately one-third of the participants in the study were found to have been diagnosed with MetS and its individual components, including hyperglycemia, low HDL, hyperlipidemia, and hypertension. Additionally, 59.92% of participants had central obesity. Participants with MetS tended to be older, Mexican American or non-Hispanic white, have higher levels of education, be married, have higher BMI, consume alcohol in moderation, be physically inactive, have higher BUN levels, and have higher levels of inflammatory index (i.e., SII, LC, NC). Supplementary Table S1 presents the characteristics of participants stratified by SII quartiles. Participants in the highest SII quartile were generally older, female, non-Hispanic white, moderate alcohol consumers, current smokers, physically inactive, had higher BMI, and lower CR levels. Notably, Participants with MetS or its components (low HDL, hypertriglyceridemia, central obesity, hypertension) showed a higher level of SII.

**Table 1:** Population characteristics by MetS status

		**MetS**		
Characteristic	**Overall**	**No**	**Yes**	***P*** ** value**
N	6999	4460	2539	
Age (years)	47.32 (16.68)	44.15 (16.59)	53.63 (14.99)	< 0.001
< 60 yr	73.94	79.52	62.82	
≥ 60 yr	26.06	20.48	37.18	
Sex (%)				0.6
Male	48.93	48.57	49.64	
Female	51.07	51.43	50.36	
Race (%)				< 0.001
Mexican American	8.55	7.81	10.03	
Non-Hispanic White	66.56	65.91	67.84	
Non-Hispanic Black	9.98	10.97	8.00	
Other Race	14.91	15.31	14.13	
Education (%)				< 0.001
Less than high school	43.49	46.49	37.53	
High school or above	56.51	53.51	62.47	
Marital status (%)				0.002
Other	45.11	46.96	41.41	
Married	54.89	53.04	58.59	
PIR				
< 1	12.37	12.12	12.85	
≥ 1	87.63	87.88	87.15	
Drinking status (%)				0.003
Mild	57.92	55.61	62.52	
Moderate	19.92	21.16	17.43	
Heavy	22.17	23.23	20.05	
Smoking behavior (%)				< 0.001
Former	24.82	21.67	31.09	
Never	57.67	60.47	52.08	
Now	17.51	17.86	16.83	
Minutes Sedentary Activity	376.01 (199.75)	369.84 (199.75)	388.29 (199.21)	0.021
Physical activity (%)				< 0.001
No	42.52	36.53	54.46	
Moderate or vigorous	57.48	63.47	45.54	
BMI (kg/m2)	29.59 (7.07)	27.55 (6.30)	33.67 (6.75)	< 0.001
Normal	27.41	38.61	5.09	
Overweight	30.85	33.50	25.58	
Obese	41.74	27.89	69.33	
Hyperglycemia (%)				< 0.001
No	67.33	81.90	38.30	
Yes	32.67	18.10	61.70	
Low HDL (%)				< 0.001
No	72.41	88.23	40.91	
Yes	27.59	11.77	59.09	
Hypertriglyceridemia (%)				< 0.001
No	64.01	81.67	28.81	
Yes	35.99	18.33	71.19	
Central obesity (%)				< 0.001
No	40.08	56.51	7.35	
Yes	59.92	43.49	92.65	
Hypertension (%)				< 0.001
No	65.04	82.23	30.78	
Yes	34.96	17.77	69.22	
CR (mg/dl)	0.84 (0.71, 0.98)	0.84 (0.71, 0.97)	0.84 (0.71, 1.00)	0.019
BUN (mg/dl)	14.00 (11.00, 17.00)	14.00 (11.00, 17.00)	15.00 (12.00, 18.00)	< 0.001
SUA(mg/dl)	5.42 (1.40)	5.20 (1.33)	5.84 (1.43)	< 0.001
SII	448.84 (323.77, 626.20)	431.21 (312.97, 601.08)	483.97 (345.54, 665.60)	< 0.001
LC	2.10 (1.70, 2.60)	2.10 (1.70, 2.50)	2.20 (1.80, 2.70)	< 0.001
NC	4.00 (3.10, 5.10)	3.80 (3.00, 4.90)	4.50 (3.50, 5.60)	< 0.001
PC	235.00 (202.00, 273.00)	234.00 (202.00, 271.00)	236.00 (201.00, 279.00)	0.2

*Abbreviations*: *PIR* poverty income ratio, *BMI* body mass index, *CR* Serum creatinine, *BUN* blood urea nitrogen, *SUA* serum uric acid, *SII* Systemic immunity-inflammation index, *LC* lymphocyte count, *NC* neutrophil count, *PC* platelet count

%, weighted proportion. Continuous variables were shown as mean (standard deviation,SD) or median (interquartile range, IQR): *P* value was calculated by weighted Student’s t-test or Mann–Whitney U test

Categorical variables were shown as percent (%). *P* value was calculated by the weighted chi-square test

Table 2 displays the relationships between the SII and MetS as well as its individual components. In the Crude Model and Model I((adjusted for age, sex), log2-SII showed positive correlations with MetS and all its components. In Model II (All variables are adjusted), log2-SII demonstrated a positive correlation with MetS (OR = 1.18; 95% CI: 1.07–1.30) and hypertension (OR = 1.22; 95% CI: 1.12–1.34). However, the associations with the other components were no longer statistically significant. Sensitivity analysis using SII as a categorical variable (quartile) yielded consistent results with the main analysis. In Model II, participants in the highest SII quartile (Q4) had a 36% higher prevalence of MetS (OR = 1.36, 95% CI: 1.10–1.70) and a 53% higher prevalence of hypertension (OR = 1.53, 95% CI: 1.22–1.92) compared to participants in the lowest SII quartile (Q1). Moreover, the P values for trends in MetS and hypertension were significant in all models. Further analysis using RCS confirmed a linear relationship between SII and MetS (*P* for nonlinearity = 0.770, Fig. 2A). Regarding each component of MetS, SII showed nonlinear relationships with hypertension, hypertriglyceridemia, low HDL, and hyperglycemia (*P* for nonlinearity < 0.05, Fig. 2B-F). Specifically, SII exhibited a J-shaped relationship with hypertension, an inverted U-shaped relationship with hypertriglyceridemia and low HDL, and a temporary plateau relationship with hyperglycemia. Additionally, SII showed a linear plateau relationship with central obesity. The results of two piecewise linear regression models are demonstrated in Table 3. When SII exceeded 8.27, the risk for hypertension increased. SII higher or lower than 9.98 was associated with a higher risk of hyperglycemia. When SII was less than 9.27, the risk of Low HDL would increase. The cut-off value of SII for hyperglycemia was 9.98. Values less than 8.72 had more risk of hypertriglyceridemia, and rather it had less risk of hypertriglyceridemia.

**Table 2:** Association between SII with MetS and its components

	**Crude model**	**Model I**	**Model II**
	**OR (95% CI) ** ***P***	**OR (95% CI) ** ***P***	**OR (95% CI) ** ***P***
**MetS**			
Log2-SII	**1.33 (1.22, 1.45) < 0.0001**	**1.32 (1.20, 1.45) < 0.0001**	**1.18 (1.07, 1.30) 0.0059**
SII quartiles			
Q1	Ref	Ref	Ref
Q2	1.12 (0.96, 1.30) 0.1499	1.15 (0.99, 1.34) 0.0815	0.99 (0.83, 1.19) 0.9430
Q3	**1.38 (1.13, 1.69) 0.0039**	**1.40 (1.12, 1.75) 0.0064**	1.08 (0.86, 1.34) 0.5195
Q4	**1.75 (1.46, 2.09) < 0.0001**	**1.77 (1.46, 2.14) < 0.0001**	**1.36 (1.10, 1.70) 0.0214**
*P* for trend	** < 0.0001**	** < 0.0001**	**0.011**
**Hypertension**			
Log2-SII	**1.22 (1.12, 1.34) 0.0001**	**1.24 (1.13, 1.36) 0.0001**	**1.22 (1.12, 1.34) 0.0010**
SII quartiles			
Q1	Ref	Ref	Ref
Q2	1.01 (0.84, 1.21) 0.9192	1.06 (0.82, 1.36) 0.6647	1.06 (0.80, 1.42) 0.6824
Q3	1.06 (0.88, 1.27) 0.5374	1.04 (0.83, 1.32) 0.7264	0.98 (0.79, 1.22) 0.8698
Q4	**1.50 (1.27, 1.78) 0.0001**	**1.59 (1.28, 1.96) 0.0002**	**1.53 (1.22, 1.92) 0.0054**
*P* for trend	** < 0.0001**	** < 0.0001**	**0.002**
**Hyperglycemia**			
Log2-SII	**1.11 (1.02, 1.21) 0.0253**	**1.13 (1.04, 1.23) 0.0078**	1.08 (1.00, 1.17) 0.0885
SII quartiles			
Q1	Ref	Ref	Ref
Q2	1.06 (0.87, 1.29) 0.5669	1.09 (0.89, 1.34) 0.3944	1.04 (0.84, 1.30) 0.7249
Q3	1.05 (0.84, 1.30) 0.6886	1.08 (0.86, 1.36) 0.5003	0.98 (0.76, 1.25) 0.8497
Q4	**1.23 (1.03, 1.47) 0.0343**	**1.28 (1.08, 1.51) 0.0095**	1.15 (0.96, 1.38) 0.1644
*P* for trend	0.054	**0.023**	0.259
**Low HDL**			
Log2-SII	**1.29 (1.14, 1.46) 0.0003**	**1.27 (1.12, 1.43) 0.0007**	1.11 (0.96, 1.28) 0.1706
SII quartiles			
Q1	Ref	Ref	Ref
Q2	1.07 (0.85, 1.35) 0.5499	1.06 (0.85, 1.33) 0.6047	0.93 (0.72, 1.20) 0.5896
Q3	**1.51 (1.15, 1.99) 0.0062**	**1.46 (1.12, 1.92) 0.0104**	1.18 (0.88, 1.59) 0.3058
Q4	**1.63 (1.27, 2.08) 0.0006**	**1.57 (1.23, 2.00) 0.0014**	1.18 (0.89, 1.56) 0.2784
*P* for trend	** < 0.001**	** < 0.001**	0.136
**Hypertriglyceridemia**			
Log2-SII	**1.15 (1.06, 1.25) 0.0020**	**1.19 (1.09, 1.30) 0.0007**	1.06 (0.95, 1.17) 0.3185
SII quartiles			
Q1	Ref	Ref	Ref
Q2	1.21 (1.00, 1.46) 0.0576	1.24 (1.02, 1.50) 0.0388	1.09 (0.89, 1.33) 0.4321
Q3	**1.41 (1.13, 1.76) 0.0056**	**1.50 (1.18, 1.89) 0.0027**	1.23 (0.96, 1.58) 0.1337
Q4	**1.31 (1.10, 1.57) 0.0063**	**1.40 (1.16, 1.69) 0.0020**	1.09 (0.88, 1.35) 0.4593
*P* for trend	**0.005**	**0.002**	0.372
**Central obesity**			
Log2-SII	**1.41 (1.26, 1.58) < 0.0001**	**1.33 (1.19, 1.50) < 0.0001**	1.12 (0.94, 1.34) 0.2177
SII quartiles			
Q1	Ref	Ref	Ref
Q2	**1.34 (1.08, 1.67) 0.0147**	**1.39 (1.10, 1.75) 0.0118**	1.21 (0.83, 1.76) 0.3433
Q3	**1.69 (1.36, 2.10) 0.0001**	**1.60 (1.26, 2.01) 0.0006**	1.03 (0.72, 1.47) 0.8920
Q4	**2.03 (1.57, 2.62) < 0.0001**	**1.86 (1.44, 2.40) 0.0001**	1.28 (0.87, 1.89) 0.2360
*P* for trend	** < 0.0001**	** < 0.0001**	0.335

*Abbreviations*: *Ref* Reference, *OR* odds ratio, *CI* confidence interval

Crude model: non-adjusted (univariate analysis); Model I: age and sex were adjusted; Model II: Model I plus BMI, Race, education, PIR, minutes sedentary activity, smoking behavior, drinking status, recreational. activity, minutes sedentary activity, BUN, SUA, and CR were adjusted

**Table 3:** Threshold effect analysis of log2-SII on components of MetS by using segmented logistic regression analysis

Infection point(SII)	OR	95% CI	*P* value
Hypertension			
< 8.27	0.84	0.69, 1.02	0.086
≥ 8.27	1.36	1.23, 1.50	< 0.001
Hypertriglyceridemia			
< 8.71	1.54	1.32, 1.81	< 0.001
≥ 8.71	0.86	0.74, 0.99	0.037
Hyperglycemia			
< 9.98	1.09	1.01, 1.18	0.02
≥ 9.98	1.9	1.11, 3.33	0.021
Low HDL			
< 9.27	1.37	1.23, 1.53	< 0.001
≥ 9.27	0.81	0.62, 1.04	0.1

*Abbreviations*: *OR* odds ratio, *CI* confidence interval

All of the models are fully adjusted (age, sex, race, PIR, education, drinking status, smoking status, BMI, physical activity, minutes sedentary activity, CR, BUN, SUA)

**Fig. 3:**
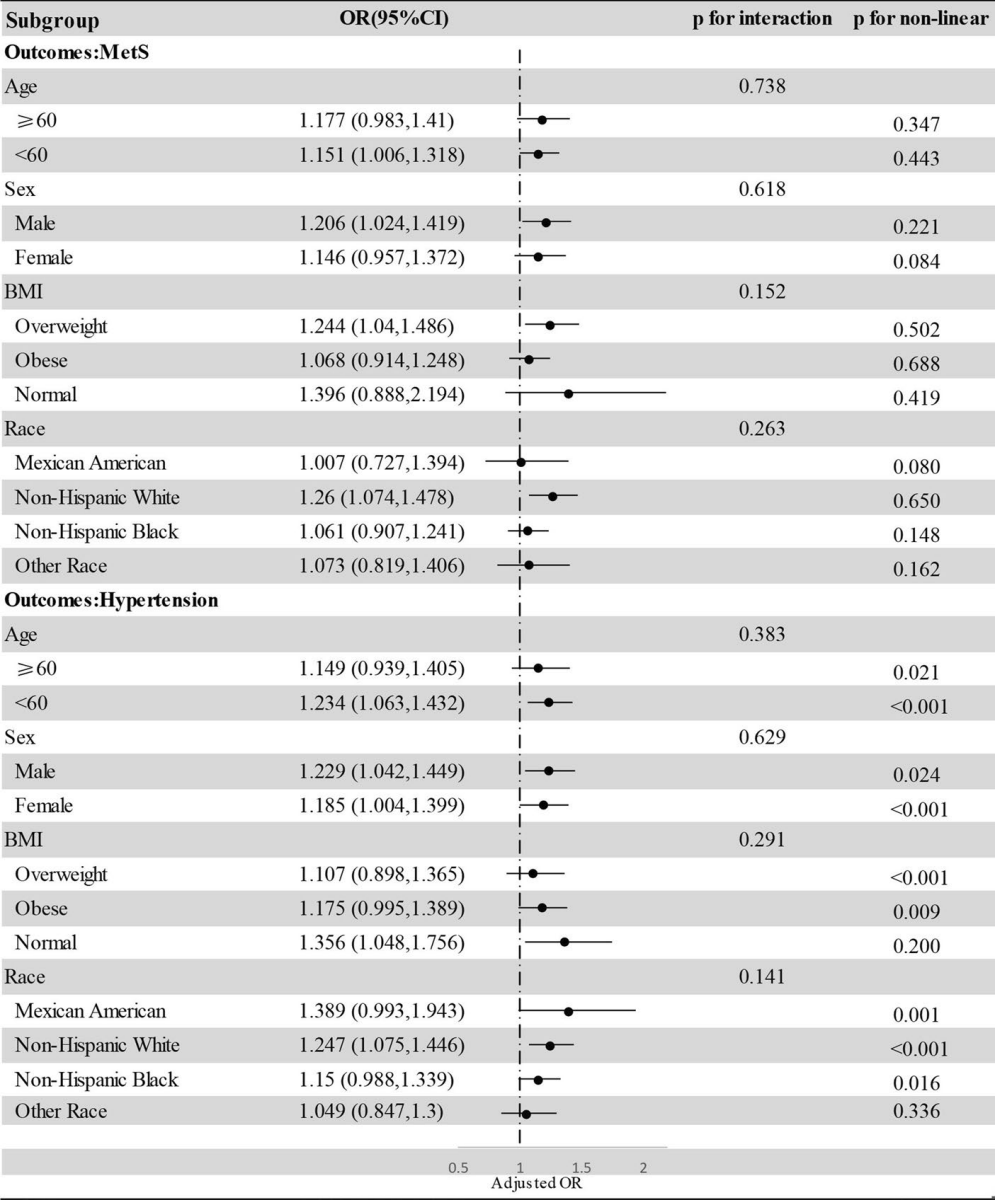
Subgroup analysis of the association of SII with MetS and hypertension

The relationship between SII and MetS and its components was investigated in Fig. 3, with particular attention given to age, sex, BMI, and race as factors for stratification. The subgroup analysis consistently revealed a specific pattern. The results revealed that there was a significant positive correlation between SII and MetS, particularly among individuals under the age of 60, male, non-Hispanic white, and overweight (*P* < 0.05, P for non-linear > 0.05). In addition, a positive and non-linear relationship between SII and hypertension was observed in subgroups consisting of individuals under the age of 60, both males and females, individuals with normal, and individuals of non-Hispanic white (*P* < 0.05, P for non-linear < 0.05). Furthermore, the interaction test revealed that these subgroups did not significantly affect the connection between SII and MetS or hypertension (*P* for interaction > 0.05). Regarding other components of MetS, hyperglycemia was positively associated with SII in individuals aged 60 years or older, females, and those of the non-Hispanic black race. Low HDL was positively associated with SII in individuals with normal BMI, central obesity was positively associated with SII in non-Hispanic black individuals, and hypertriglyceridemia was positively associated with SII in individuals with normal BMI (*P* < 0.05, P for non-linear > 0.05) (Supplementary Figure S1).

## Discussion

In the current study, a potential correlation has been identified between SII and MetS, along with its components. Through weighted logistic regression and accounting for all relevant factors, we have determined that SII is independently and positively linked to MetS and hypertension. Additionally, we have identified a significant linear relationship between SII and the risk of MetS. It is worth noting that these associations exhibit various shapes, including a J-shape, an inverted U-shape, and a temporary plateau, which correspond to hypertriglyceridemia, low HDL, and hyperglycemia respectively.

Chronic low-grade inflammation is known to cause insulin resistance, which is believed to be a key mechanism linking all components of MetS [34]. The presence of excessive free fatty acids and glucose can trigger the release of inflammatory factors, such as TNF-α, pro-inflammatory arachidonic acid, and leukotrienes, which recruit neutrophils to inflamed tissues and initiate the inflammatory response [35–38]. In metabolic disease, there is an increase in neutrophil survival and chronic accumulation at sites of inflammation, leading to prolonged release of cytokines that promote insulin resistance [39]. T regulatory cells have been found to inhibit insulin resistance and atherosclerosis by suppressing pro-inflammatory T cells and pro-inflammatory macrophages [40, 41]. HDL has the potential to exert anti-inflammatory effects through its regulation of cholesterol transport and activation of T lymphocytes [32]. Platelets, which are often highly activated in MetS and type 2 diabetes, contribute to inflammation through the release of small molecules and cytokines [42, 43], as well as by promoting the adhesion of immune cells and engaging in the process of neutrophil extracellular trap formation [44, 45]. Some drugs used to treat MetS and its components may also have anti-inflammatory effects [34]. For example, metformin inhibits Th17 inflammation in T cells through an autophagy-dependent mechanism [11], while drugs targeting the renin-angiotensin-aldosterone system suppress inflammation by inhibiting the angiotensin II-activated TLR4 cell signaling pathway and regulating inflammatory T-cell production [46, 47]. The complexity of the inflammatory response may be better reflected by SII, depending on the role of immune cells [48, 49]. Research has shown that SII is positively associated with hypertension and may predict cardiovascular mortality in hypertensive patients [50, 51]. Our findings were similar and further revealed a J-shaped relationship between SII and hypertension. SII has been also found to be a useful marker for distinguishing obese children [52]. Previous studies have reported an inverted U-shaped relationship between SII and hyperlipidemia [53]. In our research, we observed a curvilinear association between SII and various subtypes of dyslipidemia, such as low HDL and hypertriglyceridemia. Notably, SII emerged as an independent risk factor for dyslipidemia, both above and below a certain threshold.SII has also been found to have predictive value for diabetes-related complications, such as diabetic macular edema [54, 55], diabetic retinopathy [56], and depression [24]. However, the association between SII and the risk of hyperglycemia remains incompletely understood, and our study has revealed a non-linear correlation.

Our results suggest that SII may have potential clinical utility in the context of MetS. SII, as an easily obtainable and cost-effective laboratory indicator, offers several advantages. In comparison to considering a single or dual inflammatory cell type, SII reflects the interactions of three inflammatory cell types, potentially providing a more effective means of explaining the complex inflammatory mechanisms associated with MetS. In clinical practice, SII shows promise as a prospective biomarker for early screening and risk assessment of MetS. Furthermore, measuring SII levels aids in risk stratification for MetS and guides personalized clinical management and treatment effectiveness. Additionally, our study provides valuable insights for future research directions. Subsequent studies can delve into the long-term predictive and management significance of SII in the context of MetS, further validating its clinical relevance and causal relationships.

The present study possesses several notable strengths. SII has the advantage of being a non-invasive, readily available, and low-cost test method. The findings of our study offer valuable insights for future clinical practice. Our research is the inaugural investigation to examine the association between SII and MetS, along with its constituent elements, in a demographically representative cohort of American adults.SII converted into categorical variables to obtain consistent results and improve data stability. The RCS analyzed possible nonlinear relationships between SII and MetS, as well as its components. Furthermore, a stratified analysis was performed to evaluate the influence of SII.

Nevertheless, it is important to acknowledge the limitations of our study. The cross-sectional design employed in our research prevents us from establishing a causal relationship between the variables under investigation, and the potential for unmeasured confounding factors. Further information is needed through prospective studies with larger cohorts. While using the complete case method to handle missing values avoids potential estimation errors, it inevitably impacts the generalizability of the results. Furthermore, in the variable selection process aimed at enhancing the model’s interpretability, simplifying it, and preventing overfitting, we have excluded some variables, such as the use of anti-inflammatory medications, which had minimal impact on the model. This may have resulted in us overlooking the effects of some significant variables. Future research should delve deeper into these variables.

## Conclusions

Our research indicates a significant positive association between SII and both MetS and hypertension. This implies that the measurement of SII holds significant potential as a convenient and accessible indicator for the risk of developing MetS or hypertension in the general population. It is crucial to acknowledge that these findings do not establish a causal relationship. Further comprehensive prospective investigations are necessary to further authenticate these results.

### Supplementary Information

**Additional file 1: Table S1.** Population characteristics by SII quartiles. **Figure S1. **Subgroup analysis of the association of SII with other components of MetS.

## Data Availability

The study analyzed datasets that are publicly accessible. The data utilized in this study can be accessed through the following link: https://www.cdc.gov/nchs/nhanes/.

## Abbreviations

MetS: Metabolic syndrome
SII: Systemic immunity-inflammation index
NHANES: National Health and Nutrition Examination Survey
RCS: Restricted cubic spline
log2-SII: log2 transformed SII
BMI: Body mass index
HDL: High-density cholesterol
PC: Platelet count
NC: Neutrophil count
LC: Lymphocyte count
IQR: Interquartile range
SD: Standard deviation
BUN: Blood urea nitrogen
BMI: Body mass index
CR: Serum creatinine
SUA: Serum uric acid
PIR: Poverty-to-income ratio
OR: Odds ratios
CIs: Confidence intervals
