# Supplementary material for: Association of systemic immunity-inflammation index with metabolic syndrome in U.S. adult: a cross-sectional study
Source: BMC Geriatr. 2024 Jan 15;24:61. doi: 10.1186/s12877-023-04635-1 (PMC10788994; doi:10.1186/s12877-023-04635-1)
Supplement: Supplementary file 1 — Additional file 1: Table S1. Population characteristics by SII quartiles. Figure S1. Subgroup analysis of the association of SII with other components of MetS. [file 12877_2023_4635_MOESM1_ESM.docx]

| Table S1 Population characteristics by SII quartiles | | | | | | |
| --- | --- | --- | --- | --- | --- | --- |
|  |  | **SII quartiles** | | | |  |
| Characteristic | **Overall** | **Q1** | **Q2** | **Q3** | **Q4** | ***P* value** |
| N | 6999 | 1593 | 1802 | 1790 | 1815 |  |
| Age (years) | 47.32 (16.68) | 46.91 (16.56) | 46.28 (16.78) | 47.69 (16.71) | 48.35 (16.59) | 0.04 |
| <60yr | 73.94 | 74.92 | 76.21 | 72.38 | 72.35 |  |
| ≥60yr | 26.06 | 25.08 | 23.79 | 27.62 | 27.65 |  |
| Sex(%) |  |  |  |  |  | <0.001 |
| Male | 48.93 | 55.46 | 53.86 | 44.84 | 42.34 |  |
| Female | 51.07 | 44.54 | 46.14 | 55.16 | 57.66 |  |
| Race (%) |  |  |  |  |  | <0.001 |
| Mexican American | 8.55 | 7.81 | 9.67 | 8.63 | 8.01 |  |
| Non-Hispanic White | 66.56 | 58.72 | 65.56 | 68.55 | 72.47 |  |
| Non-Hispanic Black | 9.98 | 17.41 | 9.41 | 7.51 | 6.46 |  |
| Other Race | 14.91 | 16.06 | 15.37 | 15.31 | 13.06 |  |
| Education (%) |  |  |  |  |  | 0.07 |
| Less than high school | 43.49 | 46.27 | 45.32 | 40.83 | 41.87 |  |
| High school or above | 56.51 | 53.73 | 54.68 | 59.17 | 58.13 |  |
| Marital status(%) |  |  |  |  |  | 0.40 |
| Other | 45.11 | 45.84 | 43.95 | 43.43 | 47.27 |  |
| Married | 54.89 | 54.16 | 56.05 | 56.57 | 52.73 |  |
| PIR | 3.12 (1.63) | 3.12 (1.64) | 3.14 (1.65) | 3.17 (1.60) | 3.07 (1.64) | 0.7 |
| <1 | 12.37 | 12.39 | 12.74 | 11.42 | 12.91 |  |
| ≥1 | 87.63 | 87.61 | 87.26 | 88.58 | 87.09 |  |
| Drinking status(%) |  |  |  |  |  | 0.20 |
| Mild | 57.92 | 61.17 | 57.91 | 57.51 | 55.47 |  |
| Moderate | 19.92 | 17.69 | 21.32 | 20.98 | 19.42 |  |
| Heavy | 22.17 | 21.15 | 20.77 | 21.51 | 25.1 |  |
| Smoking behavior(%) |  |  |  |  |  | 0.03 |
| Former | 24.82 | 26.57 | 23.54 | 25.01 | 24.35 |  |
| Never | 57.67 | 58.59 | 60.14 | 57.71 | 54.36 |  |
| Now | 17.51 | 14.84 | 16.31 | 17.28 | 21.28 |  |
| Minutes Sedentary Activity | 376.01 (199.75) | 377.43 (197.89) | 371.26 (202.95) | 375.07 (202.91) | 380.40 (195.02) | 0.70 |
| Recreational.activity(%) |  |  |  |  |  | <0.001 |
| No | 42.52 | 35.89 | 39.25 | 46.15 | 48.01 |  |
| Moderate or Vigorous | 57.48 | 64.11 | 60.75 | 53.85 | 51.99 |  |
| BMI (kg/m2) | 29.59 (7.07) | 28.08 (6.04) | 29.03 (6.46) | 30.12 (7.19) | 30.97 (7.98) | <0.001 |
| MetS(%) |  |  |  |  |  | <0.001 |
| No | 66.58 | 72.18 | 69.88 | 65.21 | 59.74 |  |
| Yes | 33.42 | 27.82 | 30.12 | 34.79 | 40.26 |  |
| Hyperglycemia(%) |  |  |  |  |  | 0.20 |
| No | 67.33 | 69.08 | 67.83 | 68.12 | 64.52 |  |
| Yes | 32.67 | 30.92 | 32.17 | 31.88 | 35.48 |  |
| Low HDL(%) |  |  |  |  |  | <0.001 |
| No | 72.41 | 77.28 | 76.02 | 69.24 | 67.67 |  |
| Yes | 27.59 | 22.72 | 23.98 | 30.76 | 32.33 |  |
| Hypertriglyceridemia(%) |  |  |  |  |  | 0.01 |
| No | 64.01 | 68.67 | 64.47 | 60.89 | 62.53 |  |
| Yes | 35.99 | 31.33 | 35.53 | 39.11 | 37.47 |  |
| Central obesity(%) |  |  |  |  |  | <0.001 |
| No | 40.08 | 49.61 | 42.32 | 36.83 | 32.7 |  |
| Yes | 59.92 | 50.39 | 57.68 | 63.17 | 67.3 |  |
| Hypertension(%) |  |  |  |  |  | <0.001 |
| No | 65.04 | 67.85 | 67.65 | 66.58 | 58.45 |  |
| Yes | 34.96 | 32.15 | 32.35 | 33.42 | 41.55 |  |
| CR (mg/dl) | 0.84 (0.71, 0.98) | 0.87 (0.74, 1.00) | 0.85 (0.73, 1.00) | 0.83 (0.69, 0.96) | 0.81 (0.69, 0.96) | < 0.001 |
| BUN (mg/dl) | 14.00 (11.00, 17.00) | 14.00 (12.00, 17.00) | 14.00 (11.00, 17.00) | 14.00 (11.00, 17.00) | 14.00 (11.00, 17.00) | 0.32 |
| SUA(mg/dl) | 5.42 (1.40) | 5.45 (1.38) | 5.46 (1.34) | 5.36 (1.46) | 5.41 (1.41) | 0.62 |

%, weighted proportion. Continuous variables were shown as mean (standard deviation,SD) or median (interquartile range, IQR): P value was calculated by weighted Student’s t-test or Mann–Whitney U test.

Categorical variables were shown as percent (%). P value was calculated by the weighted chi-square test. Abbreviations: PIR, poverty income ratio; BMI, body mass index; CR, Serum creatinine; BUN, blood urea nitrogen; SUA, serum uric acid; SII, systemic inflammatory index;

**FigureS1** Subgroup analysis of the association of SII with other components of MetS


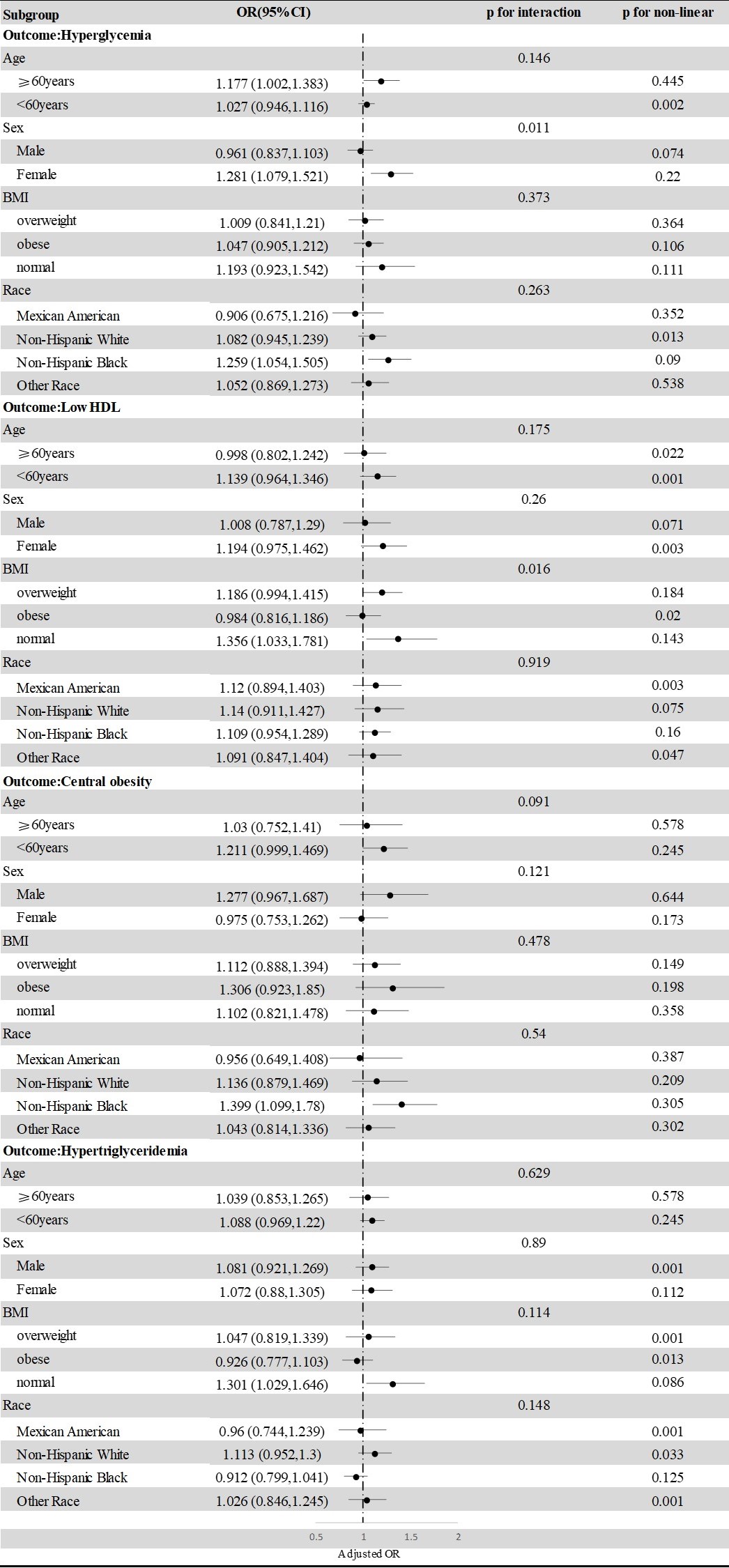


All of the models are fully adjusted (age, sex, race, PIR, education, drinking status, smoking status, BMI, physical activity, minutes sedentary activity, CR, BUN, SUA). Abbreviations: MetS, metabolic syndrome; OR, Odds ratios; CI, confidence intervals.
